# Association of Vitamin D Gene Polymorphisms With HCV Infection Outcome

**DOI:** 10.3389/bjbs.2021.10237

**Published:** 2022-03-23

**Authors:** M. Neamatallah, M. S. Serria, M. El‐Bendary, A.‐H. El‐Gilany, A. Alhawarey, S. Abed, Y. A. Setate, O. A. Ammar

**Affiliations:** ^1^ Department of Medical Biochemistry and Molecular Biology, Mansoura University, Mansoura, Egypt; ^2^ Tropical Medicine and Hepatology Department, Mansoura University, Mansoura, Egypt; ^3^ Department of Public Health and Preventive Medicine, Faculty of Medicine, Mansoura University, Mansoura, Egypt; ^4^ Infection Control Unit, Mansoura Specialized Hospital (MSH), Delta University for Science and Technology, Gamasa, Egypt; ^5^ Basic Science Department, Delta University for Science and Technology, Gamasa, Egypt

**Keywords:** HCC, VDR, polymorphism, cirrhosis, HCV

## Abstract

**Background:** Vitamin D derivatives and their receptor (VDR) are immune-response modulators in many diseases including malignancies, metabolic conditions, and infections. We hypothesized that one or more variants of *VDR* single nucleotide polymorphisms (SNPs) are associated with hepatocellular carcinoma (HCC) in hepatitis C virus (HCV) cirrhotic patients.

**Materials and Methods:** A total of 861 subjects were recruited and classified as spontaneous viral clearance (SVC, *n* = 127), chronic hepatic cirrhosis (CHC, *n* = 392), and HCC (*n* = 342). Standard routine laboratory tests were performed and clinical features noted. All individuals were genotyped for seven SNPs spanning the *VDR* using real-time PCR.

**Results:** Genotype frequencies of SNPs rs7970376, rs11568820, rs4516035, rs2228570 (Fok1), rs1544410 (Bsm-1), and rs731236 (Taq1), but not rs739837, were variously altered in CHC and HCC compared with SVC, and in HCC compared to CHC (all *p* < 0.001). The most powerful was rs7970376, which brought an OR (95% CI) of 7.14 (4.64–10.98) for HCC compared to SVC (*p* = 0.001). The carriage of the AGTAC haplotype of five SNPs were linked to CHC compared to SVC at OR 2.88 [95% CI 1.2–6.9] (*p* = 0.017) and with HCC compared to CHC at OR 1.54 [95% CI = 1.04–2.27 (*p* = 0.031).

**Conclusion:** SNPs in *VDR* may have a potential role in the outcomes of patients with HCV infection. *VDR* SNPs; rs7970376, rs11568820, rs4516035, rs2228570 (Fok1), rs1544410 (Bsm-1), and rs731236 (Taq1) could be used as molecular markers to predict the risk of HCC.

## Introduction

Hepatocellular carcinoma (HCC) is a widespread malignancy often linked to hepatitis C virus (HCV) infection, which is also a major cause of liver cirrhosis (1–3). The prevalence of HCV infection is decreasing as a result of stringent preventive regulations on blood transfusion and surgical procedures, as well as the advent of new direct-acting antiviral treatment, but rates of cirrhosis and hepatocellular carcinoma are expected to increase (4, 5). The relationship between HCV and the development of HCC is incompletely understood. Carcinogenesis is a multifactorial, dynamic phenomenon that involves both environmental and genetic and epigenetic influences (6–8). Single nucleotide polymorphisms (SNPs) play an important part in genes encoding inflammatory cytokines and growth factor ligands and receptors, including that for vitamin D (VDR). This intracellular hormone receptor affects cell growth and differentiation, embryonic development, and metabolic homeostasis by binding to the biologically active form of vitamin D. VDR is also essential for cell signaling pathways, which play a role in the development of many cancers (9).

*VDR*, at 12q12–q14, consists of eleven exons, and several SNPs been recognized, such as BsmI, ApaI, TaqI, and FokI. These have been associated with the increased risk of many tumor developments as colon (10), breast (11), prostate (12), renal cell carcinoma, and malignant melanoma (13–15). We therefore tested the hypothesis of a link between *VDR* SNPs rs7970376 (G/A), rs11568820 (A/G), rs4516035 (T/C), rs2228570 (Fok1) (C/T), rs1544410 (Bsm-1) (G/A), rs731236 (Taq1) (T/C), and rs739837 (G/T) with cirrhosis and HCC in patients with chronic HCV infection.

## Materials and Method

Our hypothesis was tested on 861 subjects admitted to the Tropical Medicine Department, and laboratory workup was conducted in the Molecular Genetic Unit in Endemic Hepatogastroenterology and Infectious Diseases, Faculty of Medicine, Mansoura University. during the period January 2016 to May 2020. All subjects gave informed written consent and the approval of all local research ethics committees. Subjects were classified into three groups. The first group of were defined as having HCV RNA level below the limit of detection in two consecutive samples taken at least 6 months apart with positive HCV antibodies in absence of a prior history of any antiviral treatment (7). Therefore, this group are described as spontaneous virus clearance (SVC). The second was defined as patients with hepatitis C infection and liver cirrhosis more than 10 years without focal lesions, denoted CHC. The third group was 342 with HCC, diagnosed when one or more liver masses ≥2 cm in diameter were detected by imaging and an AFP ≥400 ng/ml or with early arterial phase-contrast enhancement plus early venous phase contrast washout regardless of the AFP level (16).

All individuals were subjected to diagnostic work-up including laboratory liver function profile included albumin, serum total and direct bilirubin, alanine aminotransferase (ALT), aspartate aminotransferase (AST), alkaline phosphatase (ALP), international normalized ratio (INR), and tumor marker alpha-fetoprotein (AFP). Also, antinuclear antibody (ANA), serum creatinine, blood glucose, and full blood count were determined. Cirrhosis, portal vein thrombosis (PVT), presence of ascites, abdominal lymph node metastasis, splenomegaly, Barcelona clinic liver cancer (BCLC), and the Child-Pugh system were confirmed by an imaging study (abdominal ultrasonography, computed tomography, and magnetic resonance imaging).

Patients with concomitant chronic heart diseases, human immunodeficiency virus (HIV), chronic renal diseases, tumor suggestive of cholangiocarcinoma, and liver metastasis from primary tumors other than HCC (e.g. any history of alcoholism, autoimmune hepatitis, primary biliary cirrhosis, and severe nonalcoholic liver disease with metabolic syndrome) were excluded.

The Qiagen RNA extraction kit (QIA amp^®^ RNA Blood kit) (QIAGEN GmbH; Hilden, Germany) was used to extract viral RNA from serum according to the manufacturer’s instructions. Real-time PCR (Applied Biosystem, Foster City, CA, United States, 7,500 real-time PCR system) was used to assess the viral load of the collected HCV RNA samples. Seronegative HCV-Ab from the window period is pooled and centrifuged for 60 min at 15,000 rpm. The supernatant was removed, and the pellet was resuspended in 150 μL of supernatant and extracted for viral RNA assay.

Genomic DNA extraction from peripheral blood was performed for all subjects using a commercial Qiagen DNA isolation kit (QIAmp DNA Mini kit; Qiagen, Hilden’s, Germany) following the manufacturer’s instructions. A Qiagen DNA isolation kit (QIAmp DNA mini kit, Qiagen, Hilden’s, Germany) was applied to DNA extraction from peripheral blood for each individual according to the instructions of the manufacturer. The DNA was evaluated with a 2% ethidium bromide-stained agarose gel for its integrity by using a NanoDrop spectrophotometer (NanoDrop™2000/2000c, Thermo Scientific, CA, United States).

**FIGURE 1:**
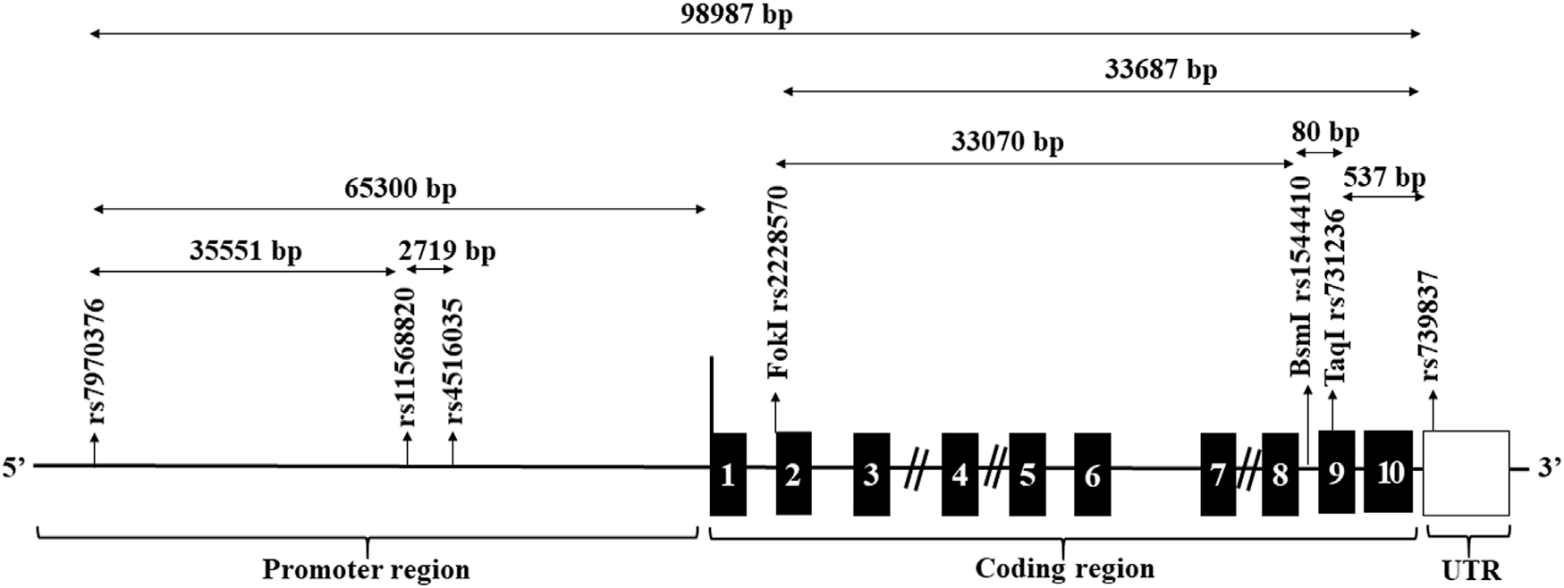
Structure of the genomic region of the *VDR* and location of candidate restriction sites. The black boxes designate the exons. The location of the candidate SNPs are arrowed.

Seven SNPs spanning the entire *VDR* were selected for the current study (Figure 1). These were three SNPs, 1- rs7970376 (G/A), 2- rs11568820 (A/G), 3- rs4516035 (T/C) in the promoter region flanking to transcriptional region, three spanning the transcriptional region; 4-rs2228570 (Fok1) (C/T), 5- rs1544410 (Bsm-1) (G/A), 6- rs731236 (Taq1) (T/C), and rs739837 (G/T) located in UTR region. SNPs were designed as primers for TaqMan allelic discrimination. The allele-specific probes were labeled with fluorescent dyes (VIC and FAM) and used in real-time PCR reaction on the apparatus (Applied Biosystems, model 7,500) for allele typing of each DNA sample using ready-made fluorescein-amidite-labeled SNP primers and probes (Applied Biosystems).

We used the following volumes of reagents for a 20 µL reaction volume: 10.0 µL of TaqMan Universal Master Mix II (2×) + 1.0 µL from SNP Genotyping Assay Mix (20×) + 1.0 µL of DNA template + 8.0 µL of RNase free water. RT-PCR genotyping was performed in a thermal cycler (Applied Biosystems, 7,500 Real-Time PCR system) with the following cycles: initial denaturation step at 95°C for 10 min, then 40 cycles of the denaturation step at 95°C for 15 s and the annealing/extension step at 60°C for 1 min. In the test plate of Taq^Man^, the fluorescence intensity of each well was read and the fluorescence data files were analyzed using automatic allele-calling software (SDS 2.4) (Applied Biosystems, Foster City, CA, United States) from each plate. Duplicated genotyped for 10 percent of all samples to ensure the quality of work. All genotyping data were exported SPSS software for statistical analysis.

Data were analyzed using the SPSS V21 (IBM Corp. Armonk, NY, United States). Categorical data were presented as numbers and percentages. Chi-square or Monte Carlo test was used for comparing the categorical data, as appropriate. The Shapiro test was used to check for normality in quantitative data. The mean and SD were used as normality distributed variables; one-way ANOVA with Bonferroni post hoc multiple comparisons was used for comparing more than two groups. The median and interquartile range were used to describe non-parametric variables. The Kruskal-Wallis test was used to compare more than two groups, whereas the Mann-Whitney test was used to compare two groups. Total genotyping of each allele was scored and counted in each group. The number of individuals who have at least one variant of a single allele is known as allele carriage. Chi-square and Fisher’s exact tests were used to compare allele carriage variables in each SNP. The odds ratio (OR) at a 95% confidence interval (CI) of a specific allele carriage was calculated, compared to that of no carriage of the target allele, using Med Calc software (Med Calc statistical software version 16.4.3). In comparison with no carriage of the target allele, the odds ratio (OR) at the 95% confidence interval (CI) of a specific allele carriage was calculated. The difference was considered significant if *p* ≤ 0.05.

## Results

The SVC group of 127 subjects were 74 males (58.3%) and 53 females (41.7%) aged 57.2 ± 5.9 years. None were diabetic or hypertensive, a smoker or drug addict, and free of any focal lesion. The CHC group of 392 were anti-HCV antibody-positive patients, of whom 264 were males (67.5%) and 127 were females (32.5%), and included 119 (30.4%) smokers. No focal lesions were found, and their mean age was 60 ± 5.1 years. The HCC group of 342 anti-HCV antibody-positive patients were 261 males (76.3%) and 81 females (23.7%), and included 128 (37.4%) smokers, and 264 (77.2%) with a focal lesion, and their mean age 60.7 ± 6.6 years (age: *p* ≤ 0.001; sex: *p* ≤ 0.001).

Table 1 shows laboratory data. As expected, there were multiple differences, many of which reflected the disease spectrum of health—cirrhosis—cancer. Table 2 shows clinical findings, also unsurprisingly more adverse in HCC. Table 3 shown allele carriage, minor allele frequencies, Hardy-Weinberg, and odd’s ratios (OR) for the SNPs. The risk of allele carriage was compared between CHC vs. SVC and HCC subjects. The frequency of all studied genotypes was all in accordance with Hardy-Weinberg genetic equilibrium in each group. Minor allele frequency ranged from 0.201 to 0.560. There was a significant risk association of the minor allele carriage of SNPs spanned promoter region and initiation codon in HCC group compared to that of both CHC and SVC groups including rs7970376; rs11568820; rs4516035; rs2228570 (Fok1); rs1544410 (Bsm-1); and rs731236 (Taq1) using either a dominant or recessive model. There was a significant association of allele carriage of the rs11568820, rs4516035, and rs1544410 (Bsm-1) in the CHC group compared to that of the SVC group. Specifically, there was a highly significant association of the rs731236 (Taq1) using either dominant or recessive models with susceptibility to CHC compared to that of the SVC group. The major association with CHC was rs731236 (Taq1) and extended to rs11568820, rs4516035, and rs1544410 (Bsm-1) compared to that of the SVC group. In addition, results indicate the protective role of rs739837 against the development of both cirrhosis and HCC.

**TABLE 1:** Laboratory characteristics of the three studied groups.

	SVC (127) mean ± SD	CHC (392) mean ± SD	HCC (342) mean ± SD	*p*
Albumin (µmol/L)	47 ± 3^A,B^	30 ± 3^A,C^	29 ± 5^B,C^	≤0.001
Total bilirubin (µmol/L)	15 (14–16)^A,B^	32 (31–36)^A,C^	68 (50–111)^B,C^	≤0.001
Direct bilirubin (µmol/L)	3 (3–3)^A,B^	14 (14–17)^A,C^	51 (36–84)^B,C^	≤0.001
ALT (µkat/L)	51 ± 11^A,B^	92 ± 19^A,C^	131 ± 59^B,C^	≤0.001
AST (µkat/L)	42 ± 9^A,B^	111 ± 29^A,C^	161 ± 071^B,C^	≤0.001
ALP (µkat/L)	19 ± 4^A,B^	21 ± 8^A,C^	191 ± 31^B,C^	≤0.001
INR	1.02 ± 0.04	1.53 ± 0.09	1.44 ± 0.19	0.07
AFP (µg/L)	2.2 (1.3–5.0)^A,B^	20.5 (13.0–42.0)^A,C^	302 (25.0–1,099.3)^B,C^	≤0.001
ANA	2.01 ± 0.01	2.01 ± 0.09	2.02 ± 0.09	1.0
Creatinine (µmol/L)	97 ± 169	109 ± 94	101 ± 22	0.1
Blood Glucose (mmol/L)	5.5 ± 0.9^A,B^	7.3 ± 1.3^A,C^	7.8 ± 1.5^B,C^	≤0.001
Platelets (x10^9^/L)	222 ± 43^A,B^	83 ± 17^A,C^	110 ± 39^B,C^	≤0.001
WBCs (x10^9^/L)	8.1 ± 1.5^A,B^	6.4 ± 3.5^A^	6.8 ± 5.9 ^B^	≤0.001
Haemoglobin (g/L)	135 ± 12^A,B^	96 ± 11^A^	95 ± 18 ^B^	≤0.001

Data mean (SD) or median with IQR, range. *p* value by ANOVA, or KW, Kruskal-Wallis test. A, B, C significant difference between corresponding groups by Bonferroni (or Mann-Whitney) post-hoc multiple comparisons, as appropriate. SVC: spontaneous virus clearance, CHC: chronic hepatic cirrhosis, HCC: hepatocellular carcinoma, ALT: alanine aminotransferase, AST: aspartate aminotransferase, ALP: alkaline phosphatase, INR: international normalized ratio, AFP: alpha-fetoprotein, ANA: antinuclear antibody, WBCs: White blood cells.

**TABLE 2:** Clinical findings characterization and classification of CHC and HCC.

	CHC (392)	HCC (342)	*p*
	N (%)	N (%)	
Cirrhosis	392 (100)	166 (48.5)	≤0.001
Portal vein thrombosis	27 (6.9)	192 (56.1)	≤0.001
Ascites	378 (96.4)	159 (46.5)	≤0.001
Spleen	57 (14.5)	166 (48.5)	≤0.001
Child-Pugh			
A	3 (0.8)	8 (2.3)	≤0.001
B	329 (86.3)	192 (56.1)	
C	49 (12.9)	142 (41.5)	

CHC: chronic hepatic cirrhosis, HCC: hepatocellular carcinoma, N: number.

**TABLE 3:** The genotype distribution of VDR SNPs in the three studied groups.

SNPS				Genotype model				MAF	HW
rs7970376	GG	GA	AA	Dominant		Recessive			χ^2^/*p*
				OR (95% CI)	*p*	OR (95% CI)	*p*		
SVC	81	33	8	R		R		0.201	
CHC	179	127	76	0.72 (0.49–1.06)^1^	0.099	0.55 (0.33–0.90)^1^	0.0167	0.365	3.02
HCC	79	165	88	7.14 (4.64–10.98)^2^	0.0001	6.64 (3.15–13.99)^2^	0.0001	0.514	0.08
				1.66 (1.23–2.22)^3^	0.0008	1.69 (1.26–2.27)^3^	0.0004		
rs11568820	AA	AG	GG						
SVC	63	49	13	R		R		0.300	
CHC	177	129	83	1.40 (0.95–2.50)^1^	0.0853	2.31 (1.26–4.26)^1^	0.0070	0.379	0.601
HCC	94	139	102	7.13 (4.64–10.98)^2^	<0.0001	2.77 (2.04–3.77)^2^	0.0001	0.512	0.46
				2.19 (1.63–2.94)^3^	<0.0001	1.82 (1.32–2.50)^3^	0.0003		
rs4516035	TT	TC	CC						
SVC	75	41	8	R		R		0.230	
CHC	232	102	57	1.23 (0.83–1.81)^1^	0.3091	2.40 (1.13–5.13)^1^	0.007	0.276	0.5408
HCC	104	131	106	4.34 (2.89–6.53)^2^	<0.0001	4.14 (1.96–8.78)^2^	0.0002	0.503	0.4620
				3.52 (2.66–4.72)^3^	<0.0001	3.10 (2.19–4.40)^3^	<0.0001		
rs2228570 (Fok1)	CC	CT	TT						
SVC	57	49	17	R		R		0.337	1.4620
CHC	180	129	76	1.07 (0.73–1.58)^1^	0.7235	1.49 (0.86–2.60)^1^	0.159	0.365	0.2266
HCC	73	152	114	3.58 (2.35–5.44)^2^	<0.0001	3.67 (2.13–6.32)^2^	<0.0001	0.560	
				3.34 (2.44–4.56)^3^	<0.0001	2.46 (1.78–3.40)^3^	<0.0001		
rs1544410 (Bsm-1)	GG	GA	AA						
SVC	53	46	22	R		R		0.372	
CHC	146	126	107	1.37 (0.93–2.03)^1^	0.1133	1.77 (1.08–2.90)^1^	0.0240	0.449	4.1977
HCC	94	109	131	2.32 (1.55–3.50)^2^	0.0001	3.05 (1.86–4.99)^2^	<0.0001	0.555	0.0404
				1.70 (1.26–2.28)^3^	0.0005	1.72 (1.28–2.32)^3^	0.0003		
rs731236 (Taq1)	TT	TC	CC						
SVC	42	68	12	R		R		0.377	4.2434
CHC	93	171	114	1.96 (1.28–3.01)^1^	0.0021	4.05 (2.17–7.55)^1^	<0.0001	0.528	0.0394
HCC	97	110	130	1.74 (1.14–2.67)^2^	0.0111	5.42 (2.91–10.10)^2^	<0.0001	0.549	
				0.89 (0.68–1.22)^3^	0.4682	1.34 (1.00–1.80)^3^	0.0518		
rs739837	TT	TG	GG						
SVC	30	60	31	R		R		0.504	
CHC	148	134	99	0.55 (0.35–0.86)^1^	0.0086	0.9 (0.57–1.40)^1^	0.6170	0.436	0.0081
HCC	120	134	81	0.81 (0.52–1.27)^2^	0.5171	0.84 (0.53–1.33)^2^	0.4547	0.442	0.92815
				1.02 (0.83–1.47)^3^	0.5171	0.94 (0.68–1.30)^3^	0.7121		

R, reference category; ^1^, CHC, vs. SVC; ^2^, HCC, vs. SVC; ^3^, HCC, vs. CHC; SNPs, single nucleotide polymorphisms; MAF, minor allele frequency; HW, Hardy-Weinberg equilibrium; OR, odds ratio; CI, confidence interval; SVC, spontaneous virus clearance; CHC, chronic cirrhosis; HCC, hepatocellular carcinoma.

To eliminate any false-positive result for the association of each individual SNP, the linkage disequilibrium was calculated using Haploview 4.2 software. The carriage of common haplotypes of the rare alleles were AGTAC (rs7970376, rs11568820, rs2228570 (Fok1), rs1544410 (Bsm-1), rs731236 (Taq1)) and ACG (rs7970376, rs4516035, rs739837). The carriage of the AGTAC haplotype was higher in CHC compared to SVC (OR = 2.88 [95% CI 1.2–6.9] *p* = 0.0175) and in HCC compared to CHC (OR 1.54 [95% CI 1.04–2.27] *p* = 0.031). Furthermore, the ACG haplotype was significantly increased in CHC (OR 4.6 [95% CI 2.07–10.25] *p* < 0.001) and HCC (OR 1.45 [95% CI 1.02–2.07] *p* = 0.038).

## Discussion

Many cases of HCC are resistant to chemotherapy and radiotherapy, and only patients with minor asymptomatic HCC have a modest long-term survival (17). Early diagnosis is desirable as it improves the prognosis, allowing resection, radiofrequency ablation, and transplantation (18, 19), especially for HCV-infected patients, who are at higher risk for HCC (20). Genetic differences may clarify the incidence of HCC caused by HCV between different populations. In addition, several susceptible genetic loci of HCC have been identified and validated.

This study showed that patients with HCC have a higher frequency of rs7970376, rs11568820, and rs4516035 SNPs in the promoter region as compared to both CHC and SVC. Furthermore, SNPs associated with HCC extended to rs2228570 (Fok1), rs1544410 (Bsm-1), and rs731236 (Taq1). Similarly, earlier studies have documented that many *VDR* SNPs have been associated with HCC (21–23). In addition, the association between *VDR* SNPs and HCC development in chronic HCV patients was reported in two studies (23, 24). However, neither VDR BsmI, ApaI, nor TaqI were associated with HCC in Taiwanese with hepatitis B carriers (25). The results of genotype distribution showed that patients with CHC had a higher frequency of rs731236 (Taq1) polymorphism in the coding region as compared to SVC subjects. Furthermore, SNPs associated with CHC extended to rs11568820, rs4516035, and rs1544410 (Bsm-1). Other studies have also shown associations between *VDR* SNPs and cirrhosis (26–30).

To increase the effect of surveillance programs, the analysis of genetics should be applied to risk modeling methods, which would allow for improved stratification and customized evaluation of optimum long-term management (31). The exact mechanism of HCC development in CHC patients, including host and viral causes, is unclear. The variations in prevalence and sex distribution in HCC are due to susceptibility variables to causative agents as well as genetic factors, in particular, inflammatory cytokine gene polymorphisms and ligands and receptor growth factors (32). In this study, the HCC patients were predominantly men *p* = 0.008 compared to the CHC group. Notably, there is a 2–7 times higher risk of HCC among men than among women, although this ratio is different worldwide (33). The reason for this may be that there are higher environmental exposures in men such as smoking and HCV infections to liver carcinogens. In this study, there were significant differences between the three groups regarding liver function tests and AFP, an established feature (18, 34). However, up to 40% of HCC patients may have normal AFP levels, particularly during the early stages (low sensitivity). In patients with cirrhosis or chronic hepatitis exacerbation higher AFP levels may be observed (low specificity) (35).

Few studies to date have investigated the associations of genetic variations of the VDR or vitamin D pathway genetic variants and cancer outcomes. *VDR* SNPs have been linked to carcinogenesis in a variety of organs, including the breast, uterus, skin, colon and rectum, and kidneys (36, 37), and have been studied in chronic liver diseases, such as chronic hepatitis B virus infections (22, 38).

VDR belongs to the nuclear receptor superfamily of ligand-inducible transcription factors, which are active in a variety of physiological processes such as cell growth and differentiation, embryonic development, metabolic homeostasis, apoptosis, and metastasis of tumor cells (22). It can also be presumed to be linked to carcinogenesis by VDR signaling pathways and the VDR gene polymorphisms.

In conclusion, our cross-sectional data support the hypothesis of links between genetic variants in *VDR* and CHC and HCC. We speculate that certain of these links have a causative role in the development of CHC and its progression to HCC, although this can only be further tested with large-scale prospective follow-up studies. If so, they may be true risk markers. The most likely candidates for such screening are rs7970376, rs11568820, and rs4516035. However, a major link with CHC was identified in the coding region rs731236 (Taq1) indicating that the alteration of VDR protein may have a potential role in the development of cirrhosis.

Our data represent a major advance in biomedical science as it shows altered frequencies of SNPs in certain regions of *VDR* in CHC and HCC, and so may be molecular markers of these two conditions.

## Summary Table

### What Is Known About This Subject?

• SNPs in *VDR* have been associated with the increased risk of many tumours.
• Polymorphisms play an important part in genes that encode inflammatory cytokines and growth factor ligands and receptors, including *VDR.*

### What This Study Adds

• *VDR* SNPs rs7970376, rs11568820, rs4516035, rs2228570 (Fok1), rs1544410 (Bsm-1), and rs731236 (Taq1) are variably linked to CHC and HCC.
• The combinations of these SNPs into haplotypes provides an additional tool in differentiating the likelihood of the presence of CHC and/or HCC.

## Data Availability

The original contributions presented in the study are included in the article/supplementary material, further inquiries can be directed to the corresponding author.
